# Charcot–Marie–Tooth Disease With Persistent Perioperative Hypotension due to Autonomic Dysfunction: A Case Report

**DOI:** 10.1155/cria/9639101

**Published:** 2025-09-29

**Authors:** Keevan Singh, Anna-Maria Lawrence

**Affiliations:** ^1^ Anaesthesia and Intensive Care Department, San Fernando General Hospital, San Fernando, Trinidad and Tobago, health.gov.tt

**Keywords:** autonomic dysfunction, Charcot–Marie–Tooth, hypotension, point-of-care ultrasound

## Abstract

Charcot–Marie–Tooth (CMT) is a hereditary neuromuscular syndrome associated with peripheral neuropathy. We describe a case of long‐standing CMT disease who underwent general anesthesia for a total knee replacement and developed significant perioperative hypotension. Clinical assessment revealed vasoplegia due to underlying autonomic dysfunction as the most likely cause. Further review also revealed a similar episode of intraoperative hypotension during a previous anesthetic. After failure of conservative strategies, the patient was started on a vasopressor infusion. However, hypotension persisted in the early postoperative period. This case serves to highlight the significance of autonomic dysfunction in CMT patients which can cause perioperative hypotension, a finding that has been rarely reported in the literature. We also discuss current diagnostic modalities that may be useful in managing cases of autonomic dysfunction that may present with hypotension.

## 1. Introduction

Charcot–Marie–Tooth (CMT) is a heterogenous hereditary condition associated with a motor‐sensory peripheral neuropathy [1]. Despite it being one of the most common inherited neuromuscular disorders, there is a paucity of literature on its perioperative considerations. Key reported issues for the anesthesiologist include the potential for prolonged neuromuscular blockade, the difficulty in monitoring neuromuscular block, and the risk of postoperative respiratory failure [1, 2]. Autonomic dysfunction with intraoperative hypotension is seldom reported.

Significant autonomic dysfunction is an uncommon clinical condition with an incidence of 1:1000 persons in the general population [3]. It is associated with labile blood pressures in response to a variety of stimuli [3, 4]. Under anesthesia with its associated reduction in both sympathetic tone and cardiovascular compensatory mechanisms, patients with efferent autonomic dysfunction can experience marked hypotension [3, 4]. Successful management of these patients undergoing surgery involves early identification, invasive monitoring, optimization of fluid status, and use of vasopressors [3, 4].

This report presents a case of unexpected and persistent intraoperative hypotension as a potential perioperative consideration in a patient with CMT disease. Potential diagnostic tools that can aid in the diagnosis are also discussed.

## 2. Case Presentation

A 55‐year‐old female presented for right total knee replacement secondary to severe osteoarthritis. Her medical history was significant for CMT which she was diagnosed more than thirty years ago. She was currently not taking any medication and reported good functional capacity limited only by her osteoarthritis. She had previous ankle arthrodesis performed under general anesthesia. Routine preoperative investigations were normal.

Standard monitors were applied, and intubation and ventilation were subsequently facilitated with 100 μg fentanyl, 100 mg propofol and 16 mg of cis‐atracurium. Her preinduction blood pressure was 140/85 mmHg. Femoral nerve block was performed under direct ultrasound guidance using 15 mLs of 0.25% Bupivacaine solution with 1:200,000 adrenaline. Immediately following induction, the patient developed severe hypotension (Mean Arterial Pressure 50–55 mmHg) with a heart rate of 60‐65bpm. Ephedrine boluses totaling 20 mg were given, followed by a 500 mL bolus of Lactated Ringers which resulted in a minimal increase in blood pressure. The concentration of sevoflurane was lowered to 1.5% while emergency point‐of‐care ultrasound (POCUS) was performed with a phased array probe (Kosmos, EchoNous, Redmond Wa).

**Figure 1:**
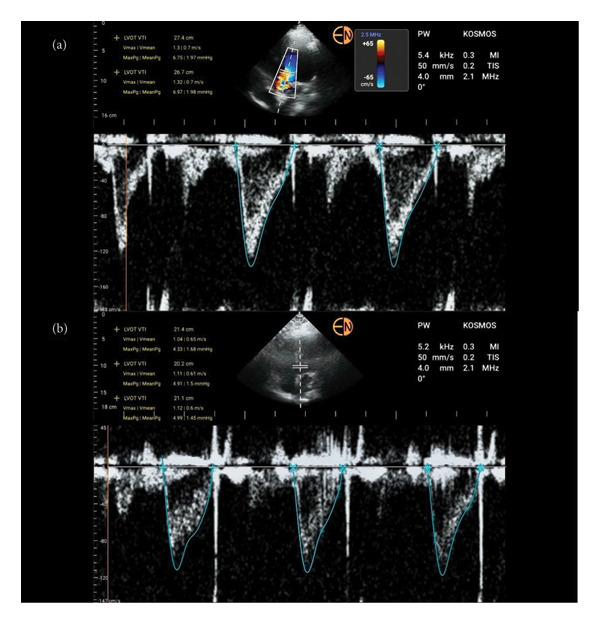
Pulse wave Doppler VTI tracings obtained from the apical five chamber view. (a) VTI obtained intraoperatively which showed an average VTI of 27 cm. (b) VTI obtained 4 hours after end of surgery which showed an average VTI of 20 cm.

POCUS views were limited to the chest wall as the subcostal region was inaccessible due to the surgical field. Thus, we were unable to visualize the Inferior Vena Cava. POCUS revealed normal left ventricular and right ventricular function with no regional wall motion abnormalities. Left Ventricular Internal Diastolic Diameter (LVIDd) was measured at 4.3 cm. Pulse wave (PW) Doppler of mitral inflow showed a E/A ratio of 1.3. Tissue Doppler of the lateral mitral wall revealed normal to low filling pressures with a E/e’ of 6.6. However, before additional fluids were given, Left Ventricular Outflow Tract Velocity Time Interval (LVOT VTI) (Figure 1(a)) was measured and found to be increased (27 cm) making hypovolemia an unlikely cause of the patient’s hypotension.

Despite the initial reduction in depth of anesthesia, the patient’s perfusion index was noted to be continuously increasing during the anesthetic (peak of 8%). These findings made us strongly suspect vasoplegia secondary to autonomic dysfunction as the most likely diagnosis. A metaraminol infusion was commenced peripherally at a rate of 1.25–2.5 mg/hr to maintain hemodynamic stability. Hemodynamics improved significantly after this with a BP of 120/60 being largely maintained for the duration of the case. Surgery lasted approximately 3 hours with a total blood loss of less than 100 mLs and a urine output of 900 mLs.

At the end of the operation, the neuromuscular block was reversed with 2.5 mg of neostigmine and 1.2 mg of atropine. Of note, acceleromyography was unable to elicit any twitches during the case, even in the preinduction calibration phase. This was taken to be likely as a result of her neuropathy. Once tidal volumes were adequate, the patient was extubated, and the metaraminol infusion was discontinued.

In the recovery room following surgery at 40° head up, the patient was again noted to be hypotensive, with systolic blood pressure being less than 100 mmHg and dipping to as low as 70 mmHg. The patient complained of slight dizziness but minimal pain. Her head was adjusted down, and another fluid bolus was given with a transient increase in blood pressure to a systolic of more than 100 mmHg. POCUS was again performed which showed a well‐contracting left ventricle, with a LVIDd of 4.7 cm, a E/A ratio of 1.2 and an LVOT VTI of 21.4 cm. Given the risk of further episodes of hypotension the patient was transferred to the intensive care unit (ICU) for monitoring and peripheral vasopressors if needed.

Four hours after surgery, POCUS now revealed a relatively decreased LVOT VTI (Figure 1(b)) of approximately 20 cm. Overnight in the ICU, patient remained comfortable with stable systolic BPs and adequate urine output.

When reviewed the subsequent day, the patient remained comfortable with isolated episodes of systolic BP < 100 mmHg and complained of mild dizziness on attempting lower limb physiotherapy. Postoperative troponin was negative. The patient was eventually discharged home hemodynamically stable 6 days later.

On postoperative questioning, our patient revealed a similar case of perioperative hypotension that occurred during and after her last anesthetic. During further questioning, she disclosed that she had forgotten to mention this to the anesthetic team, as she was unaware of its significance. On review of the previous anesthetic chart, which was unavailable preoperatively, persistent hypotension despite a relatively low anesthetic depth was confirmed.

## 3. Discussion

Autonomic dysfunction can be commonly encountered in the perioperative period in patients with uncontrolled diabetes [3]. In these patients there is significant cardiovascular lability with frequent use of vasopressors and increased lifetime mortality [5, 6]. In their review of perioperative autonomic dysfunction, Mustafa et al. did not include CMT as a causative factor for autonomic dysfunction [3]. However, Stojkovik et al. reported that autonomic dysfunction may be one of the major features associated with a certain rare CMT subtype [7]. Despite this, there are only two other reported cases of CMT‐associated autonomic dysfunction in the perioperative period [4, 8]. Similar to us, Maroun and Arin et al. both reported cases of unexpected hemodynamic instability in CMT patients which was clinically reasoned to be due to autonomic dysfunction [4, 8]. Additionally, in one of these cases a previous episode of protracted intraoperative hypotension was also noted which allowed the anaesthetic team to make appropriate preparations for the subsequent case [4].

Diagnosis of autonomic dysfunction requires a controlled setting, and specific tests that may not be readily available in the perioperative setting [3]. Recommended tests include tilt table testing, orthostatic vital signs, valsalva testing, and the hyperventilation test [3]. As these tests are usually performed in specialized centres, confirmatory testing may be lacking, and a high index of suspicion is necessary in suspected cases [3, 4]. However, orthostatic vital signs can be a feasible alternative in the preanaesthetic period when other confirmatory tests are absent and should have been performed in our case.

Intraoperative hypotension is common and is associated with an increased risk of mortality, myocardial injury, stroke, delirium and acute kidney injury [9]. The etiology of intraoperative hypotension is multifactorial, and common causes can include vasodilatation due to anesthetic drugs, hypovolemia, antihypertensive drugs, myocardial dysfunction, and autonomic dysfunction [9]. POCUS is a novel and useful imaging modality that can be used to determine the etiology of intraoperative hypotension [10]. Its intraoperative use is, however, commonly hindered by limited patient access during surgery.

Validated ultrasound protocols that can be used intraoperatively to evaluate the causes of hypotension include FATE, RUSH, and EASy protocols [10]. While providing useful information, these studies are often qualitative analyses based on B‐mode images. More advanced parameters can include evaluating Left Ventricular Diameter, trends in VTI, and mitral inflow Doppler to evaluate filling pressures of the left ventricle and avoid fluid overload. Of these parameters VTI may be one of the most useful in the management of shock [11, 12]. Regardless of the functional impairment of the myocardium of the left or right ventricle, an adequate VTI (> 20 cm) would indicate that low cardiac output is not the cause of hemodynamic instability [12].

Using PW Doppler, the Spanish Critical Care Ultrasound Network group published an advanced POCUS algorithm focused primarily on VTI for the distinguishing of the various types of shock [12]. The utility of the algorithm was demonstrated in four perioperative cases and in their subsequent rationale. A similar approach was also advocated by previous authors [12, 13]. In the patient with hypotension, a VTI of > 20 cm would indicate a primarily distributive etiology of the hypotension.

In our patient with an initial VTI of 27 cm, estimating stroke volume (SV) based on the population mean of LVOT diameter would yield a SV of almost 85 mLs [12]. Using the above rationale, this would lead us to identify the cause of the hypotension as vasoplegia. Given the clinical context and the low depth of anesthesia used, autonomic dysfunction would seem to be the major etiological factor. This would lead to an inability of the patient’s sympathetic nervous system to compensate for the vasodilation produced by the volatile anesthetic agents.

Supporting evidence for the diagnosis of vasoplegia was provided by the patient’s intraoperative perfusion index, measured on pulse oximetry. Before initiation of vasopressors and despite reduction of the depth of anesthesia, the patient’s perfusion index demonstrated an upward trend, peaking as high as 8%. This trend would have indicated reduced sympathetic tone with preserved SV [14]. Given the low dose of volatile anesthetic, this reduced sympathetic tone is not typical and would represent failure of the patient’s compensatory cardiovascular response in keeping with autonomic dysfunction. This is supported by the ultrasound findings of a high estimated SV.

Although fluid responsiveness was not assessed, given the high VTI and in the absence of signs of tissue hypoperfusion, further fluids would not be warranted even though her estimated filling pressures were low [12]. Subsequent VTI only approached a relatively normal value of 20 cm approximately 4 h after discontinuation of anesthesia which coincided with the return of hemodynamic stability.

## 4. Conclusion

Significant hypotension although scarcely reported in the literature can occur intraoperatively in CMT patients. A high index of suspicion for autonomic dysfunction should be had in patients with CMT who develop significant hypotension despite low doses of inhaled volatile anaesthetic agents.

## Consent

Written informed consent was obtained from the patient prior to manuscript submission.

## Conflicts of Interest

The authors declare no conflicts of interest.

## Author Contributions

Keevan Singh: case write up, images, manuscript write up.

Anna‐Maria Lawrence: literature review, case write up, manuscript review.

## Funding

No external source of funds was used for the publication of this case report.
